# Management of Pulmonary Hypertension in Patients on Left Ventricular Assist Device Support

**DOI:** 10.31083/j.rcm2309308

**Published:** 2022-09-13

**Authors:** Mahmoud Salem, Farah Al-Saffar, Shelley Hall

**Affiliations:** ^1^Center for Advanced Heart and Lung Diseases, Baylor University Medical Center, Dallas, TX 75246, USA; ^2^Heart and Vascular Institute, University of Pittsburgh Medical Center, Harrisburg, PA 17101, USA

**Keywords:** heart failure, pulmonary hypertension, pulmonary vasodilators, right ventricular failure, left ventricular assist device

## Abstract

Left ventricular assist devices (LVADs) are increasingly utilized for patients with end-stage heart failure (HF). Pulmonary hypertension (PH) is highly prevalent in this patient population mainly due to prolonged left ventricular (LV) failure and chronically elevated filling pressures. The effect of LVADs on pulmonary circulation and right ventricular (RV) function has recently become an area of great attention in literature. PH can lead to post-LVAD right ventricular failure (RVF) that confers a high risk of morbidity and mortality. Multiple pulmonary vasodilators, that are primarily used for the treatment of pulmonary arterial hypertension (PAH), have been studied for the treatment of PH after LVAD implantation, and some of them have shown promising results. This review aims to investigate the treatment options for PH in patients on LVADs, as well as to give an overview about the pathophysiology of PH and RVF in these patients.

## 1. Introduction 

Nearly 6.2 million adults (2.2%) in the United States are diagnosed with HF according to the National Health and Nutrition Examination Survey data [1]. End-stage HF carries a high risk of mortality. LVADs have become an important component of the treatment of end-stage HF over the past decades and were shown to improve survival in these patients. LVADs can be used as a bridge to transplantation, a bridge to recovery, or as destination therapy (DT). DT LVAD is used in patients who are not eligible for heart transplantation (HT) [2, 3, 4, 5, 6, 7, 8]. PH after LVAD implantation increases the risk of RVF and this is associated with increased postoperative morbidity and mortality [9, 10, 11, 12].

This article discusses the treatment of PH after LVAD implantation and provides an overview on the pathophysiology, the management of PH in left heart disease (LHD), and the effect of LVAD on right heart hemodynamics.

## 2. Pulmonary Hypertension in Left Ventricular Failure

PH was previously defined as elevated mean pulmonary artery pressure (mPAP) (25 mmHg or more) during right heart catheterization at rest. The cutoff has been recently reduced to 20 mmHg [13, 14, 15]. World Health Organization Group 2 PH (due to LHD) is a known complication of both heart failure with reduced ejection fraction (HFrEF) as well as heart failure with preserved ejection fraction (HFpEF). A pulmonary capillary wedge pressure (PCWP) >15 mmHg is typically required for the diagnosis of group 2 PH as it indicates passive pulmonary venous congestion. PCWP of 12–15 mmHg together with high clinical suspicion that increases with exercise can also be seen. There is no consensus in the literature on the exact prevalence of PH in HF patients given the heterogeneous diagnostic tests and criteria. However, the estimated prevalence ranges between 30 to 50%. It’s more prevalent in females, elderly, and in patients with hypertension or metabolic syndrome. With the progression of HF, the prevalence increases to about 70% in advanced HF patients [16, 17, 18, 19, 20, 21, 22, 23].

**Fig. 1.**
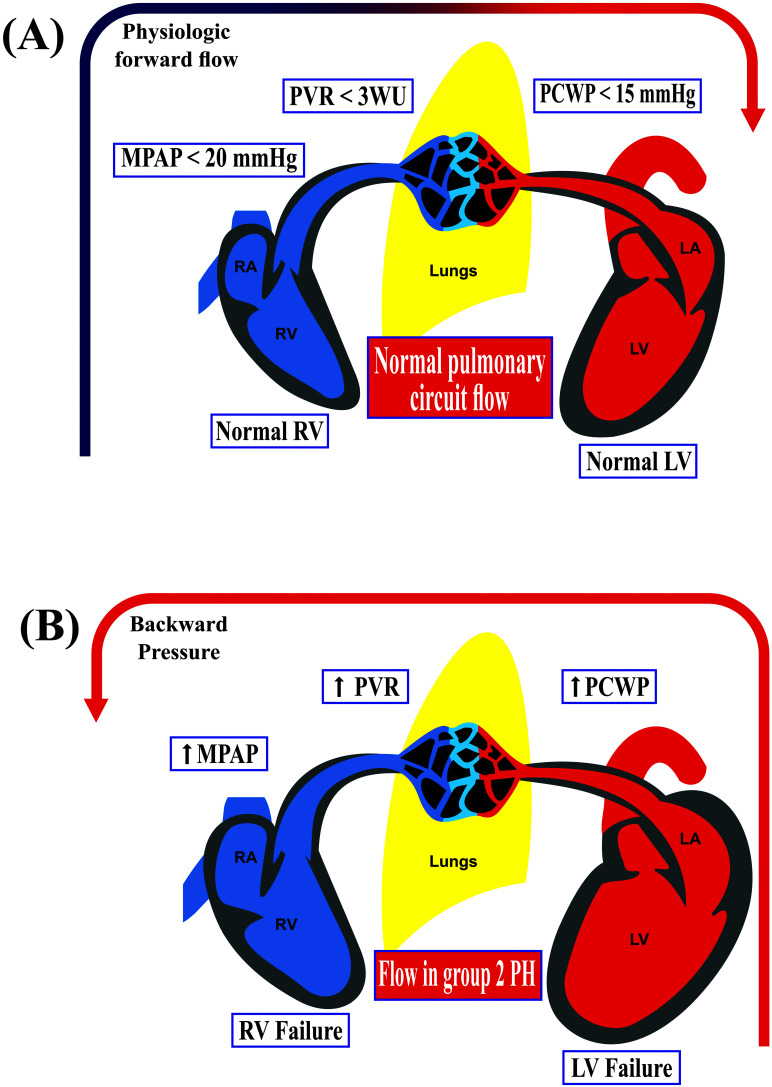
**Right heart hemodynamic consequences of left side heart failure**. (A) Physiologic flow from the right ventricle to the left side through pulmonary circulation. (B) In left side heart failure, there’s a backward pressure transmitted from elevated filling pressure to pulmonary circulation with eventual right ventricular failure. LA, left atrium; LV, left ventricle; MPAP, mean pulmonary artery pressure; PCWP, pulmonary capillary wedge pressure; PH, pulmonary hypertension; PVR, pulmonary vascular resistance; RA, right atrium; RV, right ventricle.

The pathophysiology of group 2 PH is related to elevated LA pressure that causes an increase in pulmonary venous pressure, PCWP and PH due to retrograde congestion. Additionally, prolonged pulmonary congestion leads to increased pulmonary vascular resistance (PVR) due to pulmonary vascular remodeling where pulmonary artery (PA) adapts to the afterload by an increase in wall thickness and a decrease in lumen diameter (Fig. 1). Histopathology findings in the PA include intimal fibrosis and medial hypertrophy. These changes are thought to be mediated by the imbalance between vasoactive mediators as endothelin (ET)-1 and vasodilative mediators as nitric oxide (NO) and prostacyclin. Transpulmonary pressure gradient (TPG) above 12 mmHg (which is the difference between mPAP and PCWP) indicates the presence of fixed PH. Diastolic pulmonary gradient (DPG), the difference between diastolic PA pressure and PCWP, can be used to divide group 2 PH into isolated postcapillary PH where DPG is <7 mmHg and postcapillary PH with a precapillary component where DPG is ≥7 mmHg [24, 25, 26, 27, 28, 29, 30]. The degree of PH is related to LHD severity and duration. Longstanding PH results in RVF and it is associated with poor outcomes in these patients [31, 32, 33, 34, 35, 36].

## 3. Pathophysiology of Right Ventricular Dysfunction in Patients with Left Ventricular Failure

There are anatomic and morphologic differences that exist between the RV and LV. The RV has a thinner wall and less muscular mass, mainly contracts longitudinally, unlike the LV which has strong transitional and rotational contraction forces. Normally, the RV pumps blood into a low pulmonary pressure circuit, and is able to keep output flow with an energy cost of about one-fifth of the LV which has to overcome the higher systemic pressure. Thus, the RV is more sensitive than the LV to afterload elevation and it cannot adapt to it. Additionally, there exists an interdependence between both ventricles through the interventricular septum, where LV contractility contributes nearly half of the RV work, particularly in the final twisting effect. With advanced left sided HF, elevated filling pressure transmits to the pulmonary circulation and RV. Additionally, chronic volume overload, subsequent pulmonary artery remodeling and increased PVR further increase the afterload. The RV compensates for the elevated pressure by hypertrophy, then it undergoes remodeling and dilation which eventually leads to significant reduction in the RV output and RV failure [37, 38, 39, 40, 41, 42].

## 4. Effect of LVAD on Pulmonary Artery Pressure and Right Ventricular Function

Historically, it was thought that an LVAD is contraindicated with PH associated with elevated PVR due to presumed increased risk of RVF [43]. However, the LVAD has been proven to be an effective therapy for end-stage HF and related PH. It can also improve the eligibility for HT as the chronic unloading of the LV may promote reversal of PVR elevation, thus reducing PH to an acceptable range for heart transplantation [44, 45, 46, 47, 48]. The effect of the LVAD on PA pressure and RV function has been an area of interest in the past decade [12, 49, 50]. Persistent PH after LVAD implantation increases the risk of RVF which is one of the most challenging complications of LVAD therapy, particularly early after implantation [13, 51]. Some studies have found an association between the increased preoperative PVR and the development of RVF after LVAD implantation [52], while other studies have failed to prove the same association [53, 54].

During the perioperative period, preexisting PH can be exacerbated by LVAD implantation. Possible contributing factors include the ischemia during cardiopulmonary bypass (CPB), protamine reversal, blood transfusion, physical compression of the pulmonary vessels, and pulmonary vasoconstriction due to hypercarbia and hypoxia [55]. As described above, the RV is very sensitive to changes in afterload, so postoperative PH frequently results in RVF [51]. Other mechanisms of RV failure after LVAD include volume overload from increased LV output that leads to an increased venous return and decreased RV contractility in the setting of interventricular septum leftward shift and less septal contractility. Additionally, anchoring of the LVAD to the heart apex might alter the final twisting pattern of the heart’s contraction. Acute ischemia in the perioperative period, excessive volume substitution, and arrhythmias can precipitate RVF in these patients. The prevalence of RV failure after LVAD implantation ranges from 4% to 50%. This large variation is likely related to the heterogeneity in the population characteristics and the diversity of RVF definition in the studies [50, 56, 57, 58, 59, 60, 61, 62].

Long-term, LVADs can lower PA pressure via LV unloading with resultant pulmonary decongestion [63, 64, 65, 66] but persistent elevation of PVR remains in over 40% [67, 68, 69]. Some studies on end-stage HF patients with PH who underwent LVAD implantation followed by HT reported a survival rate in these patients comparable to transplant patients with no previous PH [70, 71] albeit confounded by selection and/or survivor biases. The reduction in PA pressure should theoretically improve the long-term RV function and output, as it reduces afterload [72, 73]. However, RV dysfunction has been reported as a sequela of LVAD implantation in multiple studies due to the same mechanisms as early RVF [56, 57].

RVF after LVAD is defined by the Interagency Registry for Mechanically-Assisted Circulatory Support (INTERMACS) as elevation in central venous pressure (CVP) to >16 mmHg, as well as clinical manifestations (e.g., peripheral edema, ascites, hepatomegaly, or worsening renal/hepatic function). RVF is stratified by INTERMACS according to the duration of inotropes or vasodilators use, and the need for right-sided mechanical support. RVF is associated with a lower postoperative survival rate (59% vs. 79%) and a longer hospital stay (32 vs. 22 days). The 6 months RVF-associated mortality is 29%, and survivors suffer reduced quality of life and functional capacity [74, 75, 76, 77, 78, 79].

Of note, some studies have found that LVAD recipients who develop RVF requiring RVAD support had lower preoperative PA pressures than those who did not require RVAD support [54, 80, 81, 82] possibly due to the failing RV unable to generate high preoperative pulmonary pressures in the setting of elevated PVR [72, 83].

## 5. Treatment of Pulmonary Hypertension in Patients with Left Ventricular Failure

Multiple medical therapies have been approved for the treatment of group 1 PH or PAH (previously called primary PH) e.g., phosphodiesterase 5 inhibitors (PDE5i), ET receptor antagonists, and prostacyclins. These medications are associated with improvement in pulmonary hemodynamics, RV function and exercise tolerance [84, 85, 86, 87, 88]. The off-label use of these medications is common in group 2 PH [89]. However, there is no proven specific medications for these patients [90]. The main treatment remains managing the underlying LHD and treatment of resultant RVF (e.g., diuretics and inotropes) [91].

Due to the lack of sufficient evidence, current guidelines do not recommend any of the pulmonary vasodilators for the treatment of group 2 PH [91, 92]. A single-center study on 11 patients with LHD-PH showed that sildenafil lowers mPAP and PVR and increases cardiac output (CO) in these patients [93]. Other small studies have replicated the same findings [94, 95, 96]. The PDE5 inhibition to improve clinical status and exercise capacity in HFpEF (RELAX) trial has shown that sildenafil does not improve exercise capacity in patients with HFpEF compared to placebo. RHC hemodynamics were not measured in this trial [97].

Soluble guanylate cyclase (sGC) stimulators and ET receptor antagonists were also studied in patients with group 2 PH. Riociguat, a sGC stimulator, was shown to improve PVR, cardiac index, and stroke volume without reducing mPAP [98]. Randomized controlled trials (RCTs) did not show reduction in clinical adverse events with ET receptor antagonists (bosentan, darusentan or macitentan) [99, 100, 101, 102]. While prostacyclins are considered an acceptable treatment option for group 1 PH, A RCT on epoprostenol use in group 2 PH showed an increased mortality in these patients [103].

Severe PH in patients with LHD is considered a contraindication to isolated HT due to increased risk of RV failure, and the definitive treatment is heart-lung transplantation. Due to the limitations in donor organs, LVADs have been increasingly used for these patients [104, 105, 106, 107, 108].

## 6. Treatment of Pulmonary Hypertension after LVAD and Role of Pulmonary Vasodilators

With the increased use of LVAD for HF patients and PH, several pulmonary vasodilator drugs, including agents used for the treatment of group 1 PH, have been studied for use in post LVAD patients with persistent PH. They can be used either to wean LVAD patients from inotropes or mechanical RV support during the early postoperative period, or as long-term therapy to prevent late RVF. However, there is institutional variation in their use, and no consensus on their benefits versus risks [109]. The 2013 International Society for Heart and Lung Transplantation guidelines for mechanical circulatory support recommend the use of pulmonary vasodilators for the treatment of postoperative RVF after LVAD. The medications include NO, inhaled prostacyclin (Class I, Level of evidence C), and PDE5I (Class IIb, Level of evidence C). These recommendations are mainly based on low quality evidence from small studies and expert opinions [110]. LVAD speed should also be optimized to maximally unload the LV and this can subsequently reduce the PA pressure over time [63, 64, 65, 66].

With the assistance of an experienced librarian, we executed systematic searches of the following databases; PubMed, Ovid Medline, Embase, and Web of Science from January 1995 through December 2021. We used a combination of text words for the main concepts of HF, PH, and LVAD. Studies were limited to those including only patients above the age of 18 with HF who underwent LVAD implantation and received pulmonary vasodilator medications postoperatively. These included RCTs, prospective or retrospective observational cohort studies, and abstracts from major cardiovascular meetings.

**Fig. 2.**
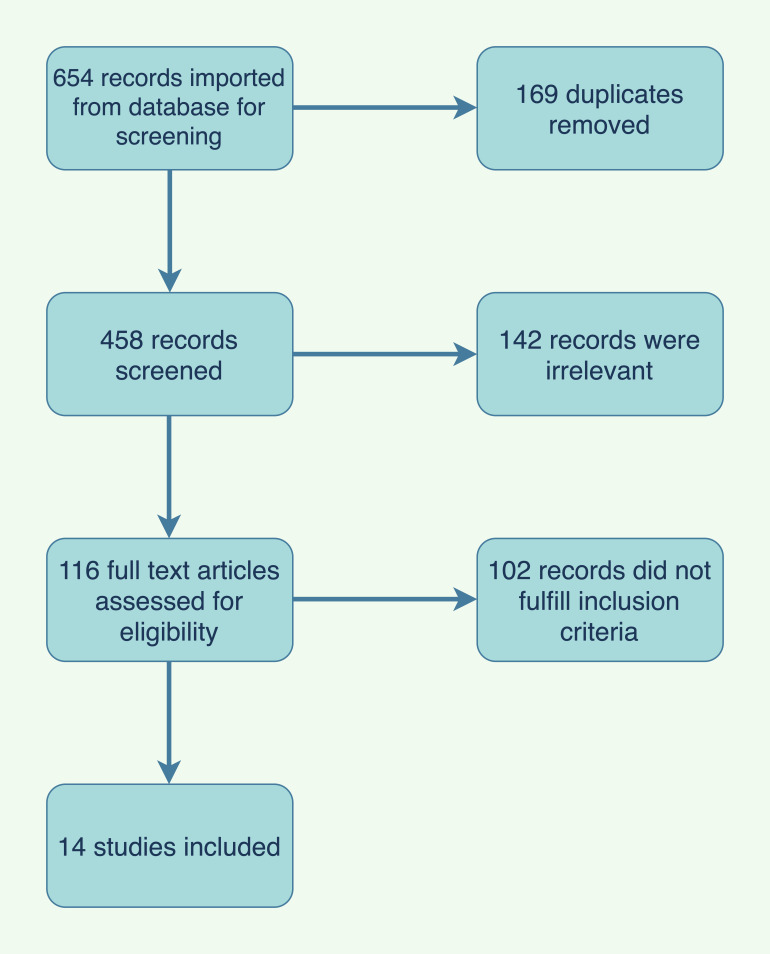
**PRISMA flowchart of pulmonary vasodilators in LVAD study selection**. PRISMA, Preferred Reporting Items for Systematic Reviews and Meta-Analyses; LVAD, left ventricular assist device.

We excluded studies that were performed in pediatrics, case reports, studies with less than 10 patients, studies conducted in animals, and studies that are not in English. All abstracts and full-text articles were maintained on the Covidence platform. Two reviewers (MS and FA) independently screened titles and abstracts to determine if they meet the inclusion criteria. The search results included 654 citations including 196 duplicates. A total of 458 were screened. 342 citations were irrelevant and 116 full articles were assessed. 13 studies were included (Fig. 2, Table 1 (Ref. [69, 111, 112, 113, 114, 115, 116, 117, 118, 119, 120, 121, 122])).

**Table 1.** **Included studies that evaluated the use of pulmonary vasodilators in patients with left ventricular assist device support**.

Study	Year	Medication	Design	Number of centers	Number of patients	Clinical setting	Main endpoint	Outcome
Argenziano *et al*. [112]	1998	NO	Prospective, randomized, double-blind	Single-center	11	LVAD insertion and elevated PVR on weaning from cardiopulmonary bypass	Postoperative hemodynamics	Inhaled NO significantly reduces mPAP and increases LVAD flow in LVAD recipients with elevated PVR.
Klodell *et al*. [111]	2007	Sildenafil	Retrospective	Single-center	10	LVADs and PH	Postoperative hemodynamics	Sildenafil reduces PA pressure, and facilitates weaning from INO and inotropes without deleterious hemodynamic consequences.
Tedford *et al*. [69]	2008	Sildenafil	Open-label clinical trial	Single-center	58	LVAD implantation, and persistent PH (defined by a PVR of 3 WU 7 to 14 days after LVAD implantation) with normal PCWP	PVR after 1–3 months	Sildenafil resulted in a significant decrease in PVR when compared with control patients.
Kukucka *et al*. [113]	2011	NO	Prospective, randomized, double-blind	Multicenter	47	PVR greater than 200 dyn 3 sec 3 cm5 before LVAD placement	Postoperative RVF	Inhaled NO was associated with reduction in PVR without effect on RV function by transesophageal echocardiography.
Potapov *et al*. [114]	2011	NO	Prospective, randomized, double-blind	Multicenter	150	Patients undergoing LVAD placement with PVR ≥200 dyne/sec/${\mathrm{cm}}^{-5}$	Postoperative RVD	Use of iNO did not achieve significance for the primary end point of reduction in RVD.
Hamdan *et al*. [115]	2014	Sildenafil	Retrospective	Single-center	14	LVAD recipients with PH and RV dysfunction prior to surgery	Postoperative hemodynamics	Perioperative sildenafil reduces mPAP and PVR and increases cardiac index in patients with PH and RVD requiring LVAD therapy.
Groves *et al*. [122]	2014	Prostacyclin	Retrospective	Single-center	37	Consecutive patients undergoing LVAD (HeartMate II) placement	Postoperative hemodynamics	Inhaled prostacyclin reduces PA pressure in the postoperative period after LVAD placement regardless of the timing of initiation.
LaRue *et al*. [120]	2015	Bosentan	Retrospective	Single-center	50	Patients with mPAP >25 mmHg	Postoperative hemodynamics and adverse events	Bosentan was associated with a decrease in PA pressure and PVR together with improvement in RV function.
Ravichandran *et al * [118]	2018	Sildenafil	Retrospective	Single-center	122	Patients undergoing LVAD implantation who survived the index hospitalization (safety)	Time to death, HF hospitalization, GI bleeding, stroke, or OHT	Sildenafil appears to be well-tolerated and safe in LVAD patients.
Gulati *et al*. [116]	2019	PDE5i	Retrospective	National registry	11,544	Continuous flow LVAD recipients	Incidence of severe early RVF	Preoperative PDE5i is associated with higher rates of post-LVAD RVF.
Xanthopoulos *et al*. [117]	2020	PDE5i	Retrospective	National registry	13,772	Continuous flow LVADs	Composite of LVAD thrombosis and ischemic stroke	PDE5i was associated with fewer thrombotic events and improved survival.
Jakstaite *et al*. [119]	2021	PDE5i	Retrospective	Single-center	109	Long-term PDE5is after discharge (safety)	Occurrence of bleeding at 12 month follow-up	PD5i is associated with increased bleeding risk.
Frantz *et al*. [121]	2021	Macitentan	RCT	Multicenter	57	MPAP ≥25 mmHg, PCWP ≤18 mmHg and PVR >3 WU	Change in PVR at week 12 of therapy from baseline	Macitentan reduced PVR in LVAD recipients and was well tolerated.

GI, gastrointestinal; LVAD, left ventricular assist device; MPAP, mean pulmonary artery pressure; NO, nitric oxide; OHT, orthotopic heart transplantation; PA, pulmonary artery; PCWP, pulmonary capillary wedge pressure; PDE5i, phosphodiesterase 5 inhibitor; PH, pulmonary hypertension; PVR, pulmonary vascular resistance; RCT, randomized controlled trial; RV, right ventricle; RVD, right ventricular dysfunction; RVF, right ventricular failure; WU, wood unit.

### 6.1 Nitric Oxide

NO is an inhaled pulmonary vasodilator that has a minimal effect on the systemic vasculature. It has been used as a first-line in the treatment of PH after LVAD placement in the operating room [111, 123, 124]. In 1998, Argenziano *et al*. [112] reported on 11 patients with LVAD and PH who were randomized to receive inhaled NO or nitrogen. NO was associated with a significant decrease in mPAP and improvement in LVAD flow. Kukucka *et al*. [113] did another RCT on the inhaled NO in LVAD patients with elevated PVR where 24 patients were assigned to inhaled NO and 23 to placebo. Inhaled NO was associated with reduction in PVR without effect on RV function by transesophageal echocardiography. On the contrary, another RCT published by Potapov *et al*. [114] did not show a significant effect of NO inhalation before separation from cardiopulmonary bypass on RV function compared to placebo. The main drawbacks of NO are the cost, the very short half-life, and the rebound effect after discontinuation [108, 111].

### 6.2 Milrinone

Milrinone is a phosphodiesterase III inhibitor that causes pulmonary vasodilation, together with its inotropic effect on the ventricles. There is paucity of data regarding the use of intravenous milrinone in LVAD recipients however it has been widely used intravenously for PH and RVF after surgery based on expert opinions and small retrospective studies [125, 126, 127, 128]. Inhaled milrinone was also found to improve hemodynamics in a small study on 10 LVAD patients [129].

### 6.3 PDE5i

PDE5i are oral pulmonary vasodilators that are used in patients with group 1 PH. Sildenafil is the most commonly used PDE5i [130]. Sildenafil has been used before and after LVAD implantation in patients with PH and/or RV dysfunction. It facilitates weaning from inotropes and NO therapy and also can overcome rebound after NO discontinuation via reduction in PA pressure and PVR [131]. Multiple retrospective studies on perioperative use of sildenafil in LVAD recipients have shown that sildenafil decreases PA pressure early after surgery. In 1998, Hamdan *et al*. [115] studied the preoperative use of sildenafil in 14 patients and the results showed a significant postoperative reduction in mPAP and PVR together with increased cardiac index. However, a national registry on pre-LVAD implantation use of sildenafil showed a higher incidence of severe postoperative RVF associated in the sildenafil group compared to the control even after propensity matching [116]. Klodell *et al*. [111] studied postoperative sildenafil administration in 10 LVAD recipients who received NO and found that sildenafil significantly reduces PA pressure and facilitates weaning from NO therapy.

Tedford *et al*. [69] performed an open-label controlled clinical trial on 58 patients to assess the effect on sildenafil on PA hemodynamics 1–3 months after LVAD implantation. Sildenafil resulted in a decrease in PVR in the treatment group with an improvement of CO and RV function. Long-term use of PDE5i was associated with fewer thrombotic events and improved survival in a national registry on 13,772 patients [117]. Another retrospective study by Ravichandran *et al*. [118] showed that sildenafil is well-tolerated and safe in LVAD patients. Only 11% of patients had to stop sildenafil due to dizziness, nausea, hypotension, resolved PH or lack of insurance coverage. On the other hand, a high bleeding risk with PDE5i was reported in a single study [119].

### 6.4 ET Receptor Antagonists

ET receptor antagonists have also been studied for patients with PH after LVAD. LaRue *et al*. [120] reported a retrospective study on the use of low-dose bosentan in 50 patients who had PH after LVAD. Bosentan was associated with decrease in PA pressure and PVR together with improvement in RV function 3–6 months after LVAD. Single RCT in 57 patients with PH showed a reduction of PVR with macitentan use early after LVAD [121].

### 6.5 Prostacyclins

Inhaled prostacyclins have been used for treatment of PAH as they reduce pulmonary pressures and improveCO [132, 133]. Small studies have reported on the use of prostacyclin after LVAD implantation [122, 134]. Groves *et al*. [122] reported a retrospective study on 37 patients and inhaled prostacyclin was found to reduce systolic and mean PA presuures in the postoperative period after LVAD placement.

## 7. Treatment of Right Ventricular Failure after LVAD

Treatment of RVF post-LVAD implantation is mainly directed toward supportive measures. Pharmacologic treatments include inotropes, diuretics to decrease preload, and pulmonary vasodilators that can reduce RV afterload by decreasing PVR as described above. Arrhythmias should be managed properly to maintain atrial-ventricular synchrony. LVAD speed should be optimized to allow proper positioning of the interventricular septum which helps RV contractility [135]. Despite medical treatment, 4–6% of patients with RVF post-LVAD are unresponsive and these patients require temporary right ventricular assist device support (RVAD) [83, 136]. Options include single lumen cannula RVAD (Biomedicus or TandemLife) or double-lumen cannula RVAD (ProtekDuo® TandemLife), other percutaneous devices (Impella RP® Abiomed), and TandemHeart (TH-RVAD). Biventricular temporary mechanical circulatory support device (veno-arterial extracorporeal membrane oxygenation) may be considered for crashing patients [137, 138]. Renal replacement therapy should be instituted for volume removal in case of concomitant renal failure and unresponsiveness to diuretics [135].

## 8. Conclusions

In conclusion, persistent PH and RVF after LVAD implantation have gained increasing interest in literature due to their significant impact on the outcome of these HF patients. Multiple pulmonary vasodilators, either immediately after surgery or in the following months, have shown therapeutic efficacy by offloading the RV, however there is paucity of supporting evidence. Further prospective RCTs are warranted to evaluate the most potent and safest options for these patients, particularly for long-term therapy.
